# Construction autophagy-related prognostic risk signature combined with clinicopathological validation analysis for survival prediction of kidney renal papillary cell carcinoma patients

**DOI:** 10.1186/s12885-021-08139-2

**Published:** 2021-04-15

**Authors:** Hongjun Fei, Songchang Chen, Chenming Xu

**Affiliations:** 1 Department of Reproductive Genetics, International Peace Maternity and Child Health Hospital, Shanghai Key Laboratory of Embryo Original Diseases, Shanghai Municipal Key Clinical Specialty, Shanghai Jiao Tong University School of Medicine, No.910, Hengshan Road, Shanghai, 200030 PR China; 2 Obstetrics and Gynecology Hospital of Fudan University, Shanghai, 200011 China

**Keywords:** Kidney renal papillary cell carcinoma, Prognostic risk signature, Autophagy-related genes, Survival prediction, Targeted therapy

## Abstract

**Background:**

Little data is available on prognostic biomarkers and effective treatment options for Kidney Renal Papillary Cell Carcinoma (KIRP) patients, to find potential prognostic biomarkers and new targets was an urgent mission for KIRP therapy.

**Methods:**

The differentially expressed autophagy-related genes (DEARGs) were screened out according to the RNA sequencing data in The Cancer Genome Atlas database, then identified survival-related DEARGs to establish a prognostic model for survival predicting of KIRP patients. Then we verified the robustness and validity of the prognostic risk model through clinicopathological data. At last, we evaluate the prognostic value of genes that formed the prognostic risk model individually.

**Results:**

We analyzed the expression of 232 autophagy-related genes (ARGs) in 289 KIRP and 32 non-tumor tissue cases, and 40 mRNAs were screened out as DEARGs. The functional and pathway enrichment analysis was done and protein–protein interaction network was constructed for all DEARGs. To sift candidate DEARGs associated with KIRP patients’ survival and create an autophagy-related risk prognostic model, univariate and multivariate Cox regression analysis were did separately. Eventually 3 desirable independent prognostic DEARGs (*P4HB*, *NRG1,* and *BIRC5*) were picked out and used for construct the autophagy-related risk model. The accuracy of the prognostic risk model for survival prediction was assessed by Kaplan–Meier plotter, receiver-operator characteristic curve, and clinicopathological correlational analyses. The prognostic value of above 3 genes was verified individually by survival analysis and expression analysis on mRNA and protein level.

**Conclusions:**

The autophagy-related prognostic model is accurate and applicable, it can predict OS independently for KIRP patients. Three independent prognostic DEARGs can benefit for facilitate personalized target treatment too.

**Supplementary Information:**

The online version contains supplementary material available at 10.1186/s12885-021-08139-2.

## Background

Renal cell carcinoma (RCC) is the sixth/eighth most common tumor in men and women. About 73,750 new cases of RCC and 14,830 RCC-related deaths happened yearly in the United States [1, 2]. Kidney Renal Papillary Cell Carcinoma (KIRP) is the second most commonly diagnosed subtype of RCC which accounting for 15–20% of RCC cases, and the most common subtype is clear cell renal carcinoma (ccRCC) [3, 4]. It has been widely assumed that KIRP has a significantly better prognosis than ccRCC in organ-confined stages [5, 6]. Most KIRP were manifested as localized disease and treated by partial nephrectomy, but substantial numbers of patients will eventually relapse [7]. About 1/3 of KIRP were manifested as metastatic KIRP (m-KIRP) [8]. Most m-KIRP patients needed systemic treatment eventually. In general, m-KIRP had a worse prognosis than metastatic ccRCC [9, 10]. As most KIRP patients had a good prognosis before metastasis, and m-KIRP patients are not so many in contrast to ccRCC, there is little data on the efficacy of available treatment options and few prognostic molecular markers have been discovered. Therefore, there is a need to explore potential prognostic markers and new molecular targets for KIRP therapy.

Traditionally, the TNM stage has been used to evaluate the risk of tumor recurrence for all RCC subtypes. However, it has limited accuracy [11]. Now, some prognostic factors such as grade and pathology stage are also used to evaluate the prognosis. However, these prognostic models were often established for ccRCC only or all RCC subtypes [12, 13]. Therefore, there is a need to refine the prognostic risk model of KIRP and build a more accurate approach for managing this second commonest subtype of RCC.

Autophagy is a non-specific, lysosome-mediated degradation. The process is beneficial for cells internally break down, clearance of damaged or superfluous proteins, and recycle cellular components. Autophagy-Related Genes (ARGs) participates in autophagy, for example, ATG7 involved in energy metabolism is an ARG. Lots of researchers proved that autophagy is associated with the progress of RCC [14–16]. For example, inhibiting autophagy in RCC increases the efficacy of many therapies [17, 18]. However, whether the expression level of ARGs has prognostic value is unknown. Hence, this research utilized ARGs to establish the prognostic risk model of KIRP. In this study, the relevance between differentially expressed autophagy-related genes (DEARGs) and clinicopathological parameters in 321 KIRP patients from TCGA database were examined, and an autophagy-related risk prognostic model was constructed as an independent predictor for overall survival of KIRP patients. We verified our risk score model from several aspects and confirmed it’s available, and we hope to provide more helpful guidance for evaluated prognosis and targeted treatment of KIRP with this novel prognostic risk model.

## Methods

### Data acquisition

There are 232 genes presently known associated with autophagy were downloaded from HADb (Human Autophagy Database, http://autophagy.lu/). The RNA-seq data and the corresponding clinical data of 289 Kidney Renal Papillary Cell Carcinoma (KIRP) patients and 32 non-tumor samples were obtained from TCGA database (The Cancer Genome Atlas database, https://www.cancer.gov/about-nci/organization/ccg/research/structural-genomics/tcga).

### DEARGs screening

The differentially expressed autophagy-related genes (DEARGs) between KIRP tissues and adjacent non-tumor tissues were identified by the Wilcoxon Rank Sum test. The filtering criteria were |log_2_FoldChange| (|log_2_FC|) > 1 and false discovery rate (FDR) < 0.05.

### *Pathway enrichment analysis and Functional annotation for* all *DEARGs*

To reveal the involved pathways and biological function of DEARGs, we performed Kyoto Encyclopedia of Genes and Genomes (KEGG) analysis and Gene Ontology (GO) analysis with the clusterProfiler package of R (version 4.0.1), and *p*-value < 0.05 was used as a strict cutoff.

### Protein–protein interaction (PPI) networks construction

The functional protein–protein interaction (PPI) analysis is performed by STRING database (Search Tool for the Retrieval of Interacting Genes, https://string-db.org/) for all DEARGs. The cut-off criterion of interaction score is 0.4. We make use of the Cytoscape software to search hub genes and achieve two-dimensional (2D) visualization of PPI networks.

### Identify the prognostic DEARGs

To identify the prognostic DEARGs whose expression profiles had notable correlation with the overall survival (OS) of patients with KIRP, the univariate Cox regression model was constructed. *P* < 0.05 is the threshold. These selected DEARGs were regarded as candidate genes had a correlation with patients’ survival.

### Prognostic risk model construction and risk score calculation

The prognostic DEARGs identified by make use of the univariate Cox regression analysis were subjected to a multivariate Cox proportional hazards model to remove the genes that might not be an independent indicator in prognosis predicting. After that, several optimal independent survival-related DEARGs were obtained and the risk score composed of expression value of these genes was established. We calculated the risk score for each patient utilizing the regression coefficients of the individual DEARGs obtained from the multivariate Cox hazards model and the expression value of each of the selected DEARGs.

$$ \mathrm{The}\ \mathrm{risk}\ \mathrm{score}=\sum \limits_{\mathrm{i}=1,2,\dots, \mathrm{n}}\mathrm{regression}\ \mathrm{coefficient}\left(\mathrm{genei}\right)\times \mathrm{expression}\ \mathrm{value}\ \mathrm{of}\ \left(\mathrm{genei}\right) $$

The risk score was calculated based on a linear combination of the relative gene expression level multiplied regression coefficients. The regression coefficients are obtained from the multiple Cox analysis and represents the relative weight of the genes. The risk score is a measure of prognostic risk for KIRP patients. Patients were divided into 2 groups by the median risk score as the critical value. High-risk score group had worse prognosis than low-risk score group.

### Assessment of prognostic risk model

To verify the robustness and validity of the prognostic risk model, we plotted the survival curves and assessed the differences in the survival rates between high-risk and low-risk groups using the log-rank test. Then, we evaluated the survival prediction accuracy of the prognostic risk model using receiver-operator characteristic (ROC) curve. The area under the curve (AUC) of ROC curve is a discrimination criterion, it ranges from 0.5 to 1.0, the higher the value, the more accurate the model.

To explore whether the autophagy-related prognostic risk model could be an independent predictor of OS not rely on other clinicopathological parameters, we performed cox proportional hazard regression analysis. The association between risk score and clinical traits were explored. To validate the prognostic value of the risk score model, we took age, sex, pathological stage, tumor grade and T classification (lymphatic metastasis excluded) as candidate risk factors for univariate and multivariate Cox regression analyses.

### Evaluation of the prognostic value of 3 prognostic-related DEARGs

As described above, to validate the availability of the prognostic risk score model, we compared the survival differences between high-risk group and low-risk group, which grouping is based on risk scores. Afterwards, we studied the association between the expression level of 3 prognostic-related DEARGs and KIRP patients’ survival individually. KIRP patients’ survival data in TCGA were used for Kaplan–Meier survival analyses.

The expression of 3 independent prognostic-related DEARGs (*P4HB*, *NRG1* and *BIRC5*) were compared and validated between normal kidney tissues and KIRP tissues in mRNA level and protein level. The mRNA expression of 3 independent prognostic-related DEARGs in kidney and KIRP tissue was analyzed utilizing cancer profiling database called Oncomine (https://www.oncomine.org/resource/main.html). The protein level of 3 independent prognostic-related DEARGs on kidney and KIRP tissue were obtained from The Human Protein Atlas database (https://www.proteinatlas.org/).

### Single-gene gene set enrichment analysis (GSEA) for 3 prognostic-related DEARGs

To explore the roles of 3 prognostic-related DEARGs in KIRP, GSEA was performed on these genes, respectively. We make use of the KEGG gene sets biological process database (version c2.KEGG.v4.0) to do GSEA. The database was affiliated with the Molecular Signatures Database (Msig DB, http://www.broad.mit.edu/gsea/msigdb/index.jsp). We exhibited 10 signal pathways containing top 5 up-regulated and top 5 downregulated signal pathways respectively, with *p* < 0.05 as a cutoff criterion. It’s worth noting that, if up-regulated pathways less than 5, we exhibit more downregulated ones instead.

## Results

### Identification of DEARGs

We downloaded the mRNA sequencing data and corresponding clinical data of 289 KIRP tissue samples and 32 normal kidney samples from TCGA database. The gene expression profile and clinical follow-up information of 265 KIRP patients were involved in our subsequent analysis. We extracted expression profile of 232 ARGs. Finally, 40 DEARGs were screened out, involved 31 upregulated ARGs and 9 downregulated ARGs with |log_2_ (FoldChange)| > 1 and FDR < 0.05 as filter criteria. The flow chart of the overall procedures in this manuscript was showed in Supplementary Fig. 1.

**Fig. 1:**
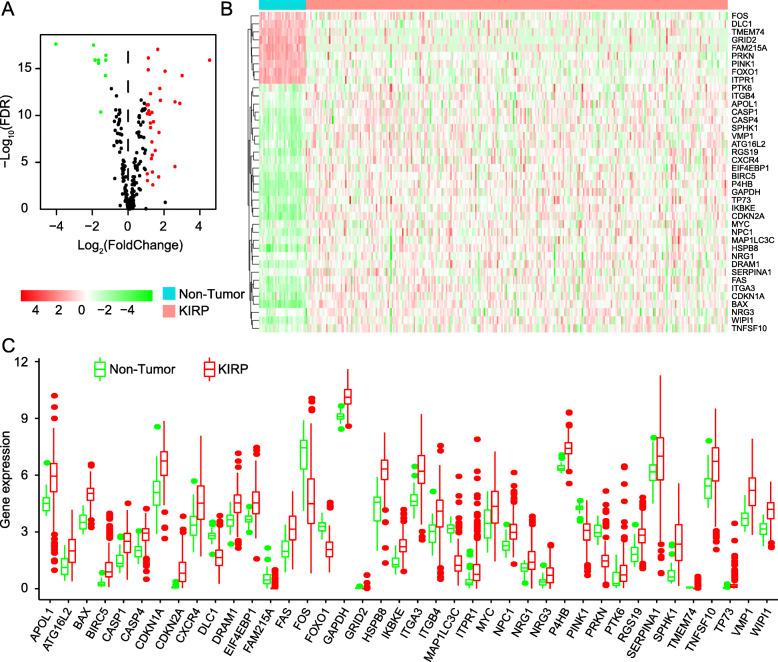
Identification of differentially expressed autophagy-related genes (DEARGs) from KIRP tissues and non-tumor kidney specimens. **a** Volcano plot of 242 autophagy-related genes (ARGs). Up-regulated genes are marked red, they are DEARGs which |Log_2_FoldChange| > 1.0 in mRNA level; Down-regulated genes are marked green and they are also DEARGs whose |Log_2_FoldChange| > 1.0. The genes whose |Log_2_FoldChange| ≤1.0 are pained black. **b** Heatmap of the expression levels of 40 DEARGs in KIRP. The color depth represents the intensity of the gene expression level. KIRP, Kidney Renal Papillary Cell Carcinoma. **c** Box plot of 40 DEARGs’ expression. Red box and green box represent KIRP or non-tumor specimens respectively

In Fig. 1a, the volcano plot exhibited the distribution of all DEARGs. X-axis of the volcano plot is log_2_FoldChange and Y-axis represents false discovery rate. The fold change patterns of 40 DEARGs in 32 non-tumor tissues and 289 KIRP tissues were showed in a heat map in Fig. 1b. The scatter plots in Fig. 1c visualized expression of 40 DEARGs between KIRP and normal tissues. In Table 1, we provide a detailed source of information on all DEARGs, including log_2_FoldChange and statistical significance.

**Table 1:** All DEARGs, screened between normal kidney tissues and KIRP tissues with criteria of FDR < 0.05 and | log_2_FoldChange| > 1

gene	Log_2_FC	*p*-Value	FDR
*PINK1*	−1.256	7.74E-19	3.91E-17
*IKBKE*	1.232429	4.06E-14	4.83E-13
*WIPI1*	1.107571	8.71E-13	7.33E-12
*FAS*	1.400674	1.10E-10	4.72E-10
*EIF4EBP1*	1.228878	1.65E-10	6.54E-10
*CDKN2A*	4.530332	4.06E-18	1.27E-16
*P4HB*	1.112831	1.86E-18	7.52E-17
*FOS*	−1.54152	7.37E-12	4.38E-11
*PTK6*	1.687081	0.000217	0.000362
*CDKN1A*	1.314733	8.48E-12	4.89E-11
*RGS19*	1.188735	1.46E-11	7.55E-11
*SPHK1*	2.867518	5.69E-13	5.23E-12
*MYC*	1.052152	9.64E-05	0.000171
*NPC1*	1.13245	5.33E-12	3.26E-11
*TMEM74*	−1.66969	1.35E-17	2.73E-16
*ITGB4*	1.32962	7.00E-07	1.72E-06
*CASP1*	1.236902	5.37E-10	2.01E-09
*GRID2*	−1.9432	3.12E-20	3.16E-18
*FAM215A*	−4.04652	1.22E-20	2.47E-18
*MAP1LC3C*	2.597344	1.42E-05	2.86E-05
*TP73*	2.60006	3.76E-13	3.62E-12
*FOXO1*	−1.2677	5.67E-18	1.27E-16
*PRKN*	−1.66938	5.48E-18	1.27E-16
*CXCR4*	1.496494	2.12E-07	5.50E-07
*VMP1*	1.752259	9.97E-15	1.34E-13
*BAX*	1.637643	1.35E-19	9.07E-18
*DLC1*	−1.28116	3.98E-16	5.75E-15
*TNFSF10*	1.281284	1.68E-06	4.09E-06
*CASP4*	1.024723	9.65E-13	7.80E-12
*SERPINA1*	1.359796	0.001698	0.002559
*HSPB8*	2.045846	1.16E-16	1.94E-15
*GAPDH*	1.105955	7.44E-17	1.37E-15
*NRG1*	1.062555	0.000569	0.000906
*NRG3*	1.341739	6.02E-05	0.000109
*ITGA3*	1.785065	2.48E-13	2.50E-12
*BIRC5*	2.995663	3.85E-16	5.75E-15
*ATG16L2*	1.310419	2.36E-08	6.70E-08
*DRAM1*	1.054383	1.18E-11	6.47E-11
*APOL1*	1.705075	1.93E-09	6.62E-09
*ITPR1*	−1.86234	5.02E-18	1.27E-16

### PPI network establishment and function annotation of all DEARGs

**Fig. 2:**
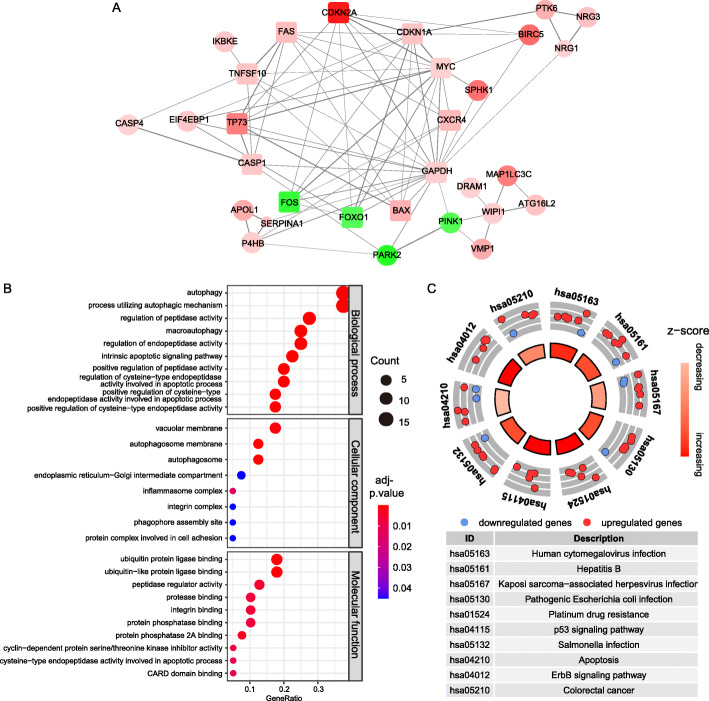
PPI network construction and functional annotation for 40 DEARGs. **a** PPI network of 40 DEARGs. The color depth of nodes is based on log_2_FoldChange, red nodes and green nodes denote up-regulated DEARGs and down-regulated DEARGs respectively. The width of links between nodes is positively correlated to combined score of protein interaction. Nodes’ size is inversely related to *p*-value. Square nodes are hub genes had most interactive protein, the number of interactive protein is > 6. **b** Gene Ontology enrichment analysis for 40 DEARGs. **c** KEGG pathway analyses for 40 DEARGs shows the top 10 signaling pathways that 40 DEARGs involved in

The interaction of all DEARGs were visualized in Fig. 2a, and there are 12 hub genes arranged in a circle are DEARGs with interaction degree > 15. Biological processes (BP) annotation reminded us that DEARGs had a strong association with enzyme-related process, such as autophagy, regulation of peptidase activity and macroautophagy. In the aspect of the molecular function (MF), DEARGs seems played vital roles in some protein binding related functions, for example, peptidase regulator activity, ubiquitin protein ligase binding and protease binding. Regarding the cellular components (CC), the DEARGs encoded proteins constituted autophagosome membrane, vacuolar membrane, endoplasmic reticulum-Golgi intermediate compartment and so on (Fig. 2b). In Fig. 2c, the mainly pathways that had a positive relation with screened DEARGs was showed, including hepatitis B, pathogenic *Escherichia coli* infection, human cytomegalovirus infection and Kaposi sarcoma-associated herpesvirus infection. Z-scores of enriched pathways were all > 0, it means that all the pathways were more likely to be enhanced.

### Establishment of autophagy-related risk signature

**Fig. 3:**
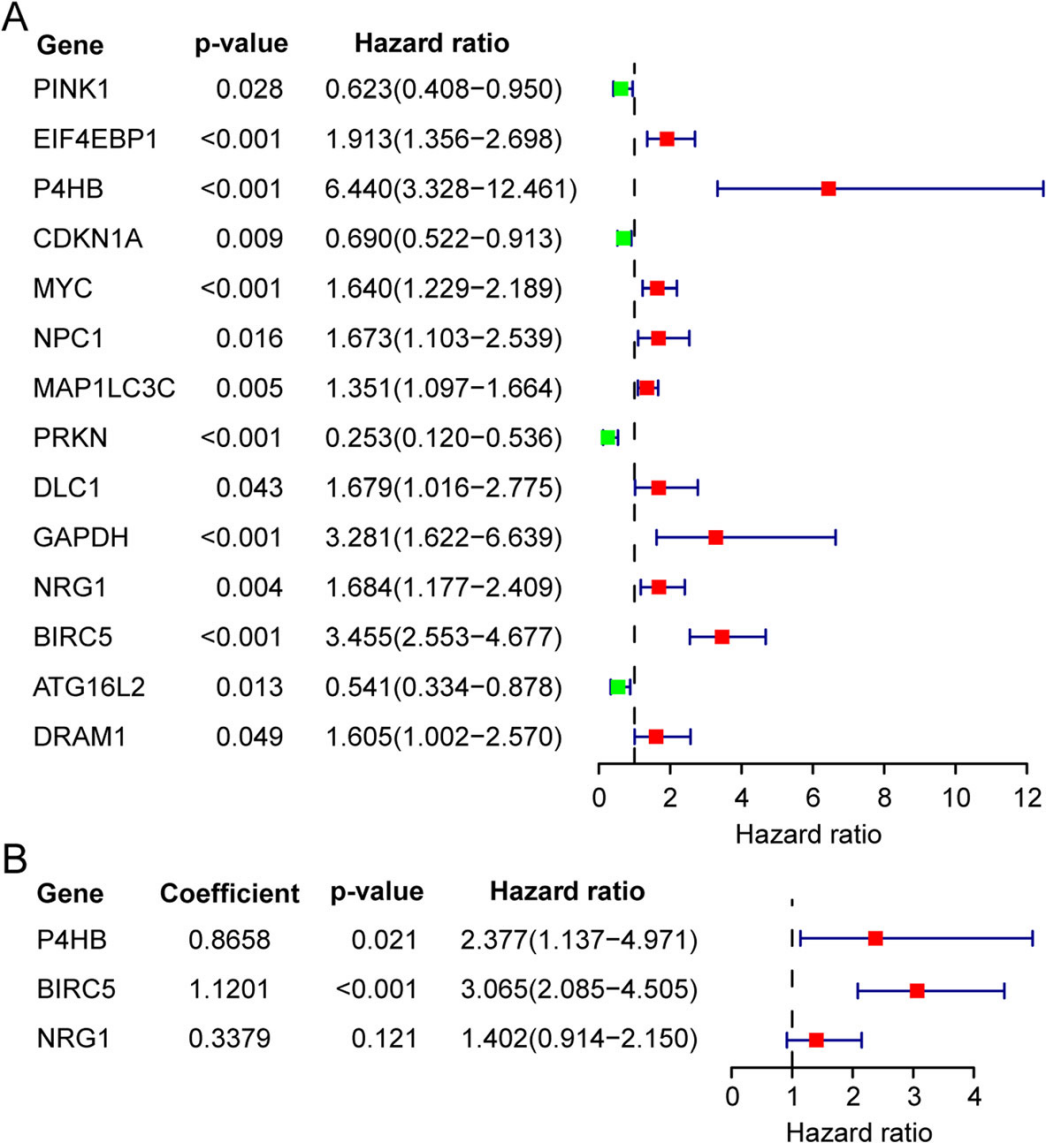
Identify survival related autophagy genes in KIRP patients and development of prognostic model. We make use of univariate and multivariate cox model to filtered out DEARGs whose expression had positive relation with KIRP patients’ survival. **a** 14 DEARGs are associated with survival of KIRP patients according to univariate cox model. **b** 3 DEARGs are significantly related to survival of KIRP patients based on multivariate cox model, we exhibited the regression coefficients and *p* values

To identify the relationship between the expression of 40 DEARGs and overall survival in KIRP patients, we constructed the univariate Cox proportional hazards model. The results showed that there are 14 DEARGs significantly related to the prognosis of KIRP patients (*p* < 0.05) (Fig. 3a). In order to raise the robustness, the screened 14 prognostic-related DEARGs were further included in the subsequent multivariate Cox regression analysis. At last, 3 DEARGs (*P4HB*, *NRG1* and *BIRC5*) were filtered out and used for autophagy-related risk model construction (Fig. 3b). The risk score for each patient was calculated according to the following formula: risk score = (0.8658× expression value of *P4HB*) + (0.3379× expression value of *NRG1*) + (1.1201× expression value of *BIRC5*). Patients were divided into high-risk (*n* = 132) and low-risk group (*n* = 133) by the median risk score as the critical value. The risk score was calculated for each patient and list of they belong to low or high-risk group (Supplementary Table 1).

### Validation of the risk signature

**Fig. 4:**
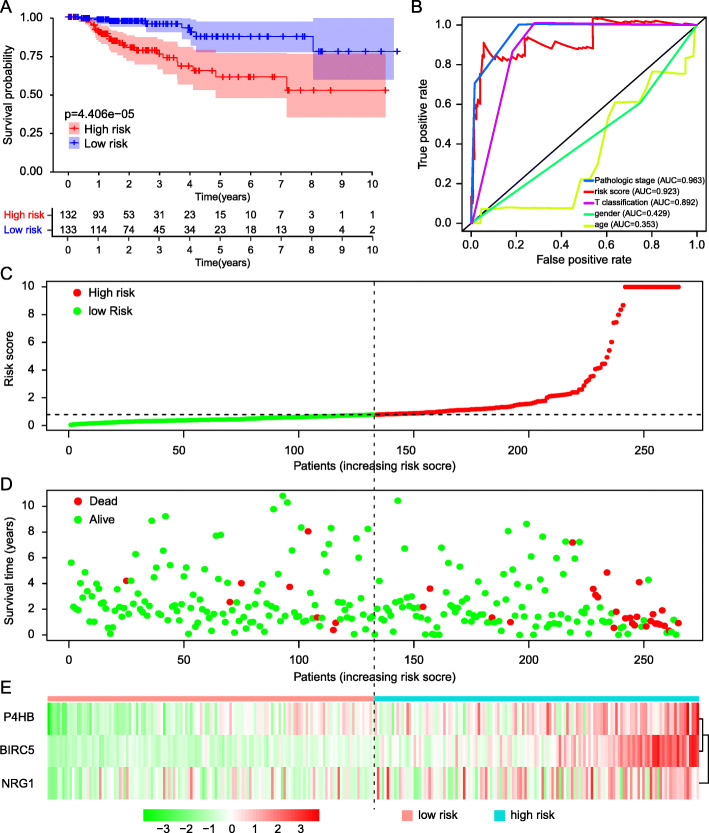
Validation of the prognostic model. **a** Kaplan-Meier plotter showed the survival probability in different risk group for KIRP patients. **b** ROC curves evaluated the accuracy of DEARGs-based risk scores for prognosis predicting. **c** Scatter plot of KIRP patients distributed as risk scores increase. **d** Scatter plot exhibited the survival status of KIRP patients as risk scores increase, red plots and green plots represents non-survivors and survivors. **e** Heatmap of 3 prognostic DEARGs which composed prognostic model as risk scores increase

To measure the accuracy of the autophagy-related risk model to predict the prognosis of KIRP patients, we draw Kaplan-Meier plotter to compare the survival time difference between high-risk and low-risk group. Low-risk group patients had more survival probability (*p* = 4.406E-05) (Fig. 4a). Next, the ROC curves were employed to determine the predictive performance of the prognostic risk model. In Fig. 4b showed, the AUC value of risk score was 0.923, it was larger than AUC values of other indicators except for the pathologic stage, which confirmed that the autophagy-related prognostic model is an excellent and independent prognostic predictor comparing with other clinicopathology indicators. The risk scores of all KIRP patients were visualized from small to large (Fig. 4c). With the increase of the risk score, the death number of KIRP patients is more (Fig. 4d). The expression patterns of 3 prognostic-related DEARGs in different risk groups was exhibited in the heatmap (Fig. 4e).

### Clinical verification of prognostic model

**Fig. 5:**
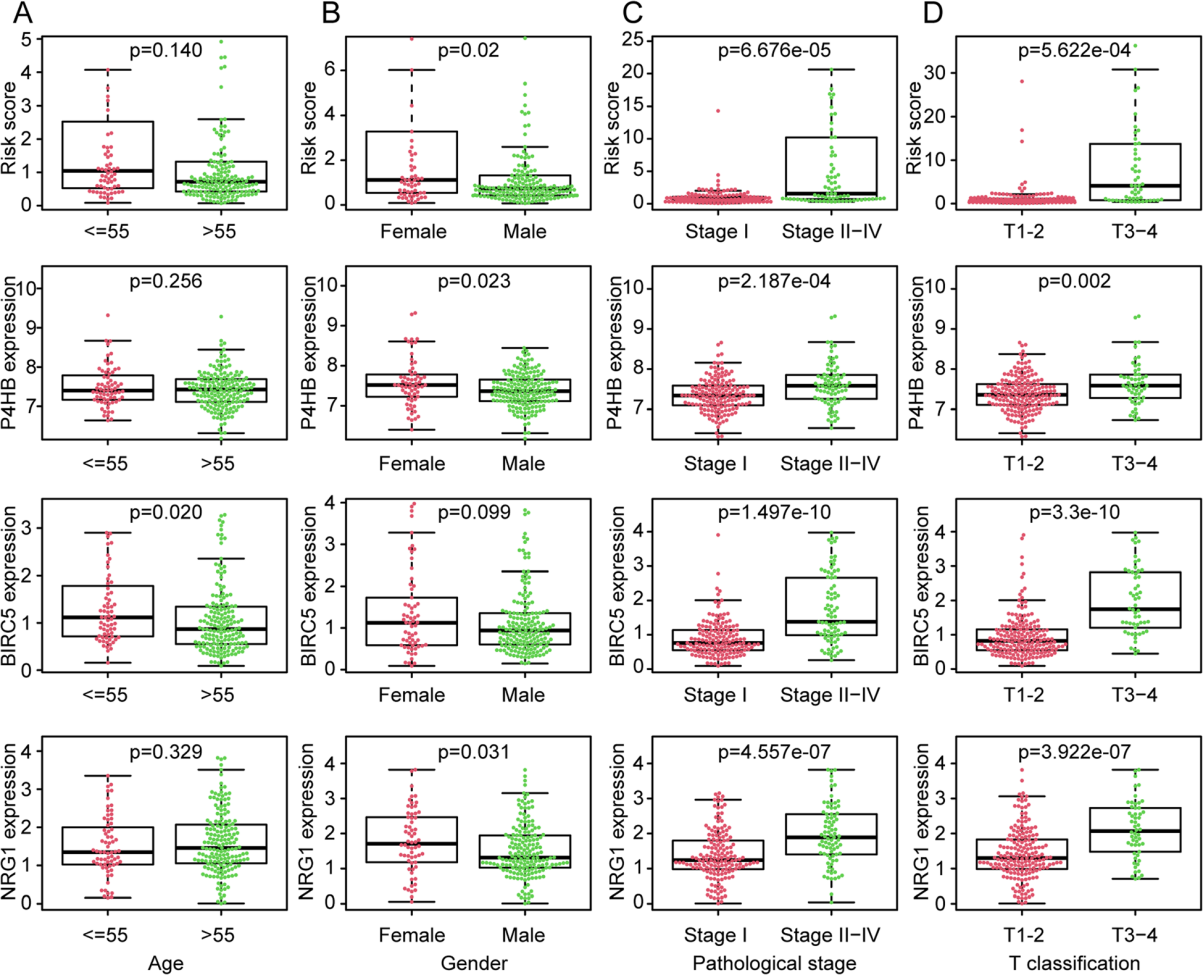
Clinical correlations between clinicopathological variables and the risk score. The relationship between expression of 3 prognostic DEARGs and clinicopathological features was also showed here. **a** Grade. **b** sex. **c** pathological stage. **d** T classification

To determine the relationship between the autophagy-related prognostic risk model and clinicopathological features in KIPR patients, we put several familiar clinicopathological factors and risk score to do univariate and multivariate cox regression analyses (Fig. 5). Since AUC values are often used to assess the performance of an individual clinicopathological indicator, and the larger of the AUC value, the more accurate of the indicator to predict prognosis. In our study, the AUC values of the clinicopathological features including age and sex to predict OS is less than 0.5 (Fig. 4b), demonstrated that age or sex alone was unable to predict prognosis as an individual indicator. The relationship between risk scores and age/sex was listed in Fig. 5a and Fig. 5b. No difference in risk score was observed between elder patients and younger patients (Fig. 5a). Instead, the AUC values of the clinicopathological features (pathological stage) and risk score is more than 0.9 (Fig. 4b), illustrated that no matter pathological stage or risk score can make a comparatively accurate prediction KIRP patients’ prognosis. Risk scores were lower in pathological stage I than in pathological stage II-IV (*p* = 6.676e-05) (Fig. 5c), and lower in T classification T1–2 than in T3–4 (*p* = 5.622e-04) (Fig. 5d). The relationship between the expression of 3 genes composed the risk model and 4 clinicopathological features in KIRP are exhibited in Fig. 5a, b, c, d. The raw TCGA data that containing basic information of all KIRP patients was listed in Supplementary Table 2. In Table 2, pathological stage, T classification, and risk score had obviously positive correlation with prognosis of KIRP patients in univariate Cox analysis, in addition, risk score and pathological stage were independent prognostic predictor of KIRP patients in multivariate Cox analysis. All above results demonstrate that the autophagy-related risk signature can be an excellent prognostic predictor ifor KIRP patients.

**Table 2:** Univariate and multivariate cox regression analyses of riskscore and clinicopathologic features in the TCGA group KIRP patients

Variables	Univariate analysis		Multivariate analysis	
	HR (95% CI)	*p*-Value	HR (95% CI)	*p*-Value
RiskScore	1.041(1.028–1.054)	**< 0.001**	1.020(1.004–1.037)	**0.016**
Age	0.986(0.956–1.016)	0.360	0.985(0.954–1.016)	0.342
Sex	0.694(0.320–1.505)	0.355	1.248(0.499–3.123)	0.636
Pathologic Stage	3.430(2.352–5.003)	**< 0.001**	3.673(2.131–6.330)	**< 0.001**
T classification	2.844(1.920–4.210)	**< 0.001**	0.786(0.430–1.438)	0.435

### Validation of the function of 3 prognostic-related DEARGs in KIRP

**Fig. 6:**
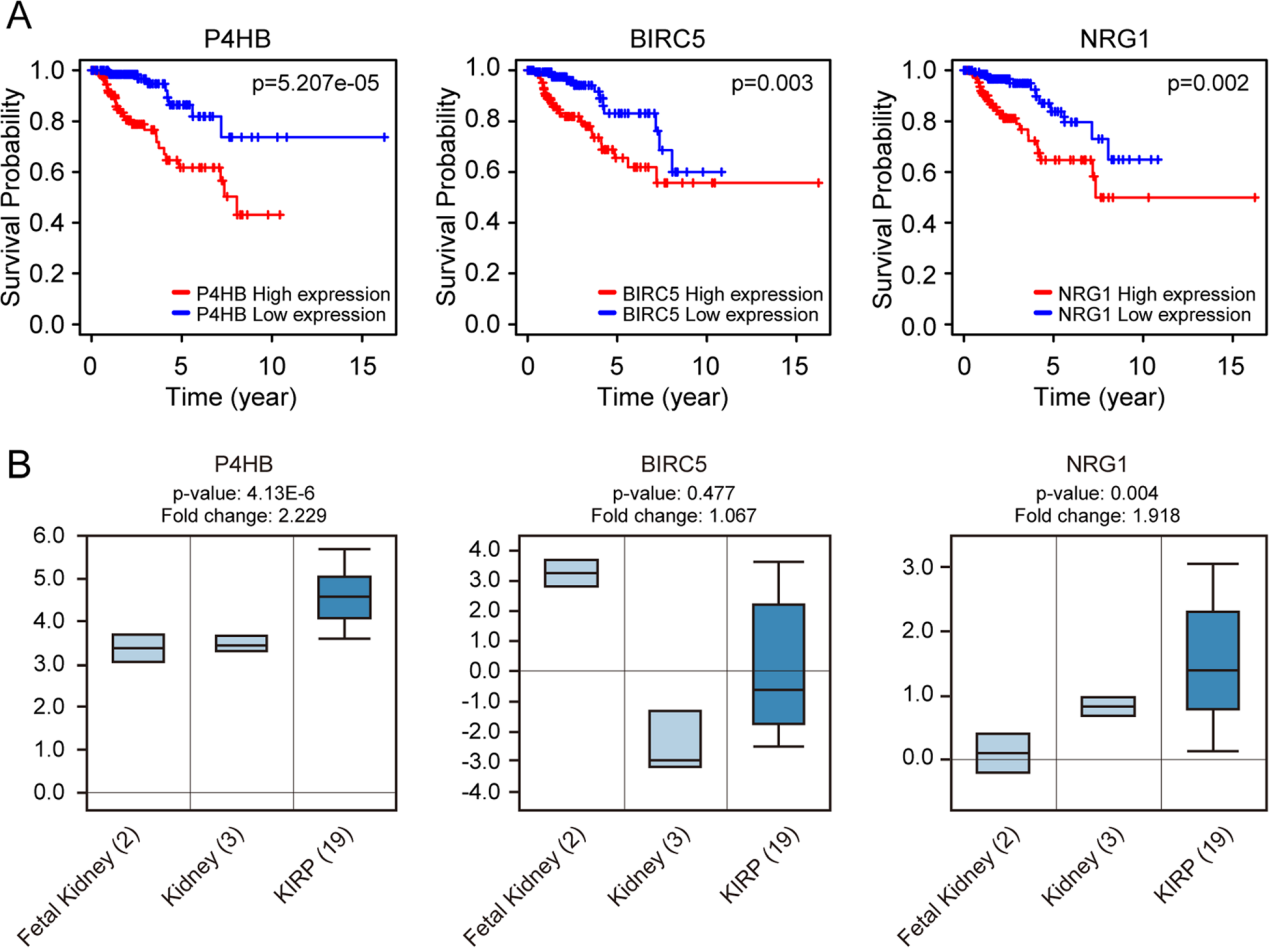
The correlation between 3 genes (*P4HB, BIRC5* and *NRG1*) identified in prognostic signature and KIRP patients’ survival (**a**). Comparing expressions of 3 genes (*P4HB, BIRC5* and *NRG1*) between normal kidney and KIRP tissues in Oncomine database (**b**)

**Fig. 7:**
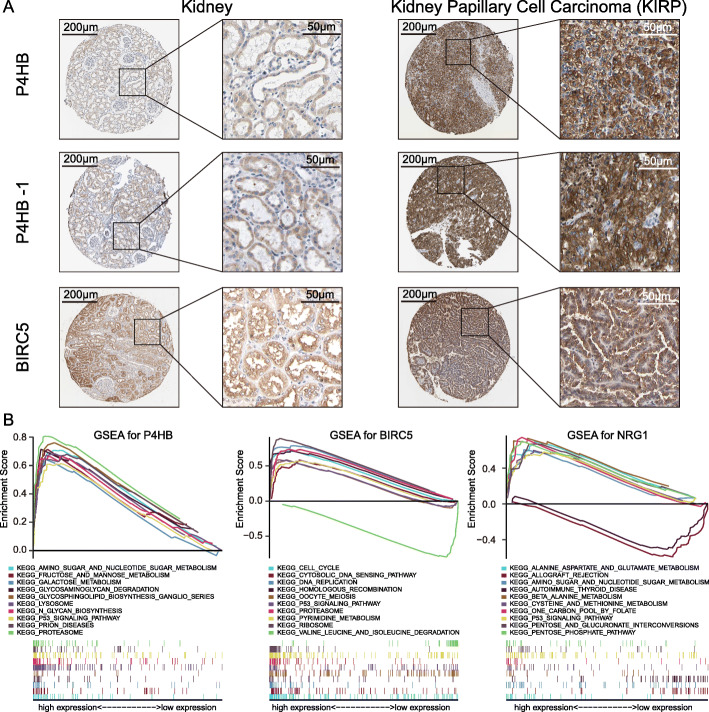
Immunohistochemistry analysis of 3 genes (*P4HB*, *NRG1* and *BIRC5*) which used to develop the risk prognostic model (**a**). *P4HB* protein is detected by antibody CAB012463 and HPA018884. *BIRC5* protein is detected by antibody HPA002830. Immunohistochemistry of *NRG1* in database of The Human Protein Atlas is absence. **b** Results of single-gene GSEA of 3 prognostic DEARGs which composed the risk signature in KIRP

According to our results, 3 prognostic-related DEARGs including *P4HB*, *NRG1* and *BIRC5* were identified to develop the survival-related risk prognostic model. We analyzed the correlation between 3 prognostic-related DEARGs that composed the prognostic model and survival probability of KIRP patients. The results of the Kaplan-Meier analysis showed that the upregulation of *P4HB* was obviously associated with the low survival probability of KIRP patients. Also, *NRG1* or *BIRC5* overexpression leads to worse OS (Fig. 6a). To further compare the expression difference of 3 prognostic-related DEARGs between KIRP and normal tissues, we performed a clinical study using cancer microarray database of Oncomine and human proteome database Human Protein Atlas. The mRNA level of 3 prognostic-related DEARGs was verified and showed in Fig. 6b. The expression trend of 3 prognostic-related DEARGs is in accordance with our previous results obtained from TCGA database which showed in Fig. 1. In Fig. 7a, results of immunohistochemistry (IHC) confirmed the expression of *P4HB*, *NRG1* and *BIRC5* protein are stronger in KIRP tissues than normal kidney tissues. Single-gene GSEA of the 3 prognostic-related DEARGs explored the potential roles of 3 prognostic DEARGs in KIRP (Fig. 7b).

## Discussion

Autophagy is a dynamic and conserved process that can maintain cellular homeostasis [19]. Many researchers had proved autophagy played an important function in cancer [20–22]. Some targeted agents aimed at autophagy had been applied in the clinics in RCC patients [23–25]. Therefore, we decided to develop an autophagy-related prognostic risk model for prognosis predicting of KIRP patients.

The autophagy-related prognostic model is comprised of 3 genes, including *P4HB*, *BIRC5*, and *NRG1*. The 3 genes are all closely relevant to clinicopathological features of KIRP patients, the expression of them is either correlated with pathological stage or T classification of KIRP patients (*p* < 0.05). The risk score obtained according to the prognostic model is also significantly associated with clinicopathological features of KIRP patients. Several assessment methods confirmed the autophagy-related signature can be an independently prognosis predictor for KIRP patients. Individual assessment of the function of 3 prognostic-related DEARGs in KIRP further proof *P4HB*, *BIRC5*, and *NRG1* are up-regulated in KIRP, and overexpression of them is associated with worse survival of KIRP patients. All results proved that risk signature constructed through *P4HB*, *BIRC5*, and *NRG1* to evaluate the prognosis of KIRP patients is clinically practicable.

*P4HB* encoded protein disulfide isomerase, it is an autophagy-related gene, Xie et al proved that *P4HB* was a novel biomarker for ccRCC diagnosis and prognosis predicting [26]. *BIRC5* also called survivin, the encoding products of *BIRC5* play a significant role in negative regulation of apoptosis or programmed cell death. Philipp et al demonstrated that *BIRC5* is of importance for renal pathophysiology and pathology [27]. *NRG1* is a growth factor of the epidermal growth factor family, the relationship between *NRG1* and RCC had not been explained clearly, but Sushma et al thought that *NRG1* fusion is a low frequency event in most tumor types, including RCC [28]. We found that most of prognostic molecular indicators and therapeutic targets were identified and verified in ccRCC only or all RCC subtypes, there is an urgent need to explore possible prognostic molecular indicators and novel targets for KIRP therapy.

Functional enrichment analysis exhibited that 40 DEARGs were mainly involved in infection related pathway. It is generally acknowledged that infection and inflammation are all correlated with carcinogenesis. Kaymakcalan et al reported that RCC patients treated with mTOR inhibitors had a risk of infection [29], Alexander et al reported that urinary tract infection history is positively associated with RCC development [30]. Hence, the affection of infection to KIRP patients should be carefully assessed and managed.

This research constructed a novel prognostic risk model for prognosis predicting of KIRP patients, and proved it is steady and credible by verification with molecular signature combined with clinical features. Several assessment methods confirmed the prognostic risk model is obviously an independently prognosis predictor for KIRP patients. We believe, apart from traditional clinicopathological features (including pathologic stage, T classification and so on), risk score derived from the autophagy-related genes signature could also be incorporated into the clinical evaluation indicators to better predict clinical outcomes. Individual assessment of the function of 3 prognostic-related DEARGs in KIRP further proved *P4HB*, *BIRC5*, and *NRG1* all play significant roles in KIRP. The 3 prognostic-related DEARGs can benefit personalized target therapy also. All results proved that the risk score calculated according to expression level of *P4HB*, *BIRC5*, and *NRG1* to evaluate the prognosis of KIRP patients is reliable.

## Conclusions

This research analyzed mRNA sequencing data of 289 KIRP tissue specimens and 32 non-tumor specimens and assessed of 232 ARGs’ expression difference in the two groups. We screened out 9 down-regulated DEARGs and 31 up-regulated DEARGs in KIRP with the threshold of |log_2_FC| > 1.0 and *P* < 0.05. From 40 DEARGs, 3 prognostic DEARGs (*P4HB*, *NRG1*, *BIRC5*) were determined to establish a prognostic risk model, and the risk score was calculated according to expression of the 3 prognostic DEARGs and fixed regression coefficients. With verification analysis combined using molecular signature and clinical characteristics, the risk score for prognosis predicting of KIRP patients is robustly and accuracy. The genes identified in autophagy-related prognostic model had been verified, and they were all correlated with KIRP patients’ prognosis, and they were all up-regulated in KIRP tissues. What’s more, this research is benefit for illustrating the molecular mechanisms behind KIRP from a new perspective.

## Supplementary Information

**Additional file 1: Figure S1.** The flow chart of the overall process in our manuscript.

**Additional file 2.**

**Additional file 3.**

## Data Availability

There are 232 autophagy-related genes downloaded from HADb (Human Autophagy Database, http://autophagy.lu/). The RNA-seq data and the corresponding clinical data of 289 Kidney Renal Papillary Cell Carcinoma (KIRP) patients and 32 non-tumor samples were obtained from TCGA database (The Cancer Genome Atlas database, https://www.cancer.gov/about-nci/organization/ccg/research/structural-genomics/tcga). The protein level of 3 independent prognostic-related DEARGs on kidney and KIRP tissue were obtained from The Human Protein Atlas database (https://www.proteinatlas.org/).

## Abbreviations

ARGs: Autophagy- related genes
AUC: Area under the curve
ccRCC: Clear cell renal carcinoma
DEARGs: Differentially expressed autophagy- related genes
DEGs: Differentially expressed genes
FDR: False discovery rate
GO: Gene Ontology
KEGG: Kyoto Encyclopedia of Genes and Genomes
KIRP: Kidney Renal Papillary Cell Carcinoma
KM plotter: Kaplan-Meier plotter
log_2_FC: Log_2_Fold Change/Logarithm of Fold Change
OS: Overall survival
PPI: Protein-protein interaction
RCC: Renal cell carcinoma
ROC: Receiver-operator characteristic
TCGA: The Cancer Genome Atlas
