# Supplementary figures and images for: Construction autophagy-related prognostic risk signature combined with clinicopathological validation analysis for survival prediction of kidney renal papillary cell carcinoma patients
Source: BMC Cancer. 2021 Apr 15;21:411. doi: 10.1186/s12885-021-08139-2 (PMC8048278; doi:10.1186/s12885-021-08139-2)

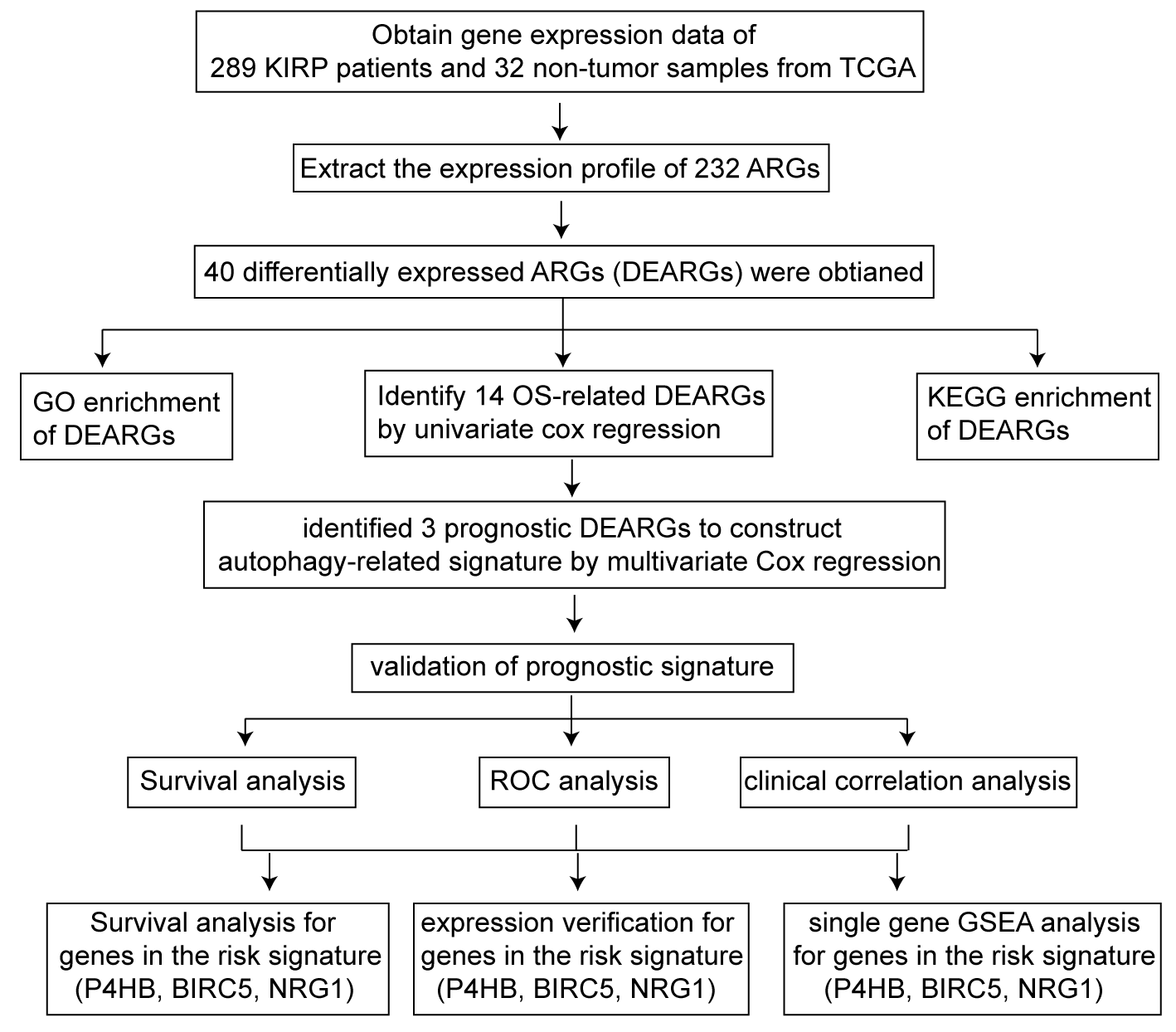

Supplement: Supplementary file 1 — Additional file 1: Figure S1. The flow chart of the overall process in our manuscript. [file 12885_2021_8139_MOESM1_ESM.tif]
